# Reliability and validity evaluation of the stigma of loneliness scale in Chinese college students

**DOI:** 10.1186/s12889-024-17738-0

**Published:** 2024-01-20

**Authors:** Zhiguang Fan, Xiaoli Shi, Shuhan Yang, Yueliang Sun, Ri Chen

**Affiliations:** 1 Department of Psychology, Shaoxing University, Shaoxing, 312000 China; 2 School of Education, Jilin International Studies University, Changchun, 130117 China; 3 School of Chinese Medicine, Changchun University of Chinese Medicine, Changchun, 130117 China; 4 School of Basic Medicine, Changchun University of Chinese Medicine, Changchun, 130117 China

**Keywords:** Stigma of loneliness, Self-stigma of loneliness, Public stigma of loneliness, Reliability, Validity

## Abstract

**Background:**

The stigma of loneliness exacerbates the negative effect of loneliness, reduces the willingness to seek help, damages interpersonal relationships, and threatens health status. However, there is currently no valid scale for measuring the stigma of loneliness in China. The study aims to translate the Stigma of Loneliness Scale (SLS) and evaluate the reliability and validity of the Chinese version.

**Methods:**

The investigation was conducted in two phases. In the first phase, the SLS was used to conduct a questionnaire survey on 657 college students aged 17 to 24; in the second phase, the SLS, the UCLA Loneliness Scale (ULS-8), the Distress Disclosure Index (DDI), the Revised Cheek and Buss Shyness Scale (RCBS), the Self-Concealment Scale (SCS), the Social Interaction Anxiety Scale (SIAS), the Social Phobia Scale (SPS), the Kessler Psychological Distress Scale (K10), and the Rosenberg Self-Esteem Scale (RSES) were used to conduct the questionnaire survey on 801 college and graduates students aged 18 to 39.

**Results:**

Two dimensions of Self-stigma of Loneliness and Public Stigma of Loneliness were extracted with a cumulative factor interpretation rate of 74.60% when conducting exploratory factor analysis on the first-stage data. The factor loading of each item ranged from 0.585 to 0.890, and the commonality ranged from 0.609 to 0.735. The confirmatory factor analysis and reliability and validity test were carried out on the data gathered in the second phase, indicating that the two-factor model fits well. In addition, the scores of SLS and all dimensions were significantly positively correlated with the total scores of ULS-8, RCBS, SCS, SIAS, SPS, and K10, and negatively correlated with those of DDI and RSES. The Cronbach’s alpha coefficients for SLS and SSL and PSL dimensions were 0.957, 0.941, and 0.955. The cross-group invariance test found that the SLS was equivalent for males and females. Meanwhile, males scored significantly higher than females on both the total scores of SLS score and each dimension.

**Conclusions:**

The Chinese version of SLS displayed satisfactory psychometric properties and can be a valid tool to assess the stigma of loneliness among Chinese young people.

Establishing and maintaining intimate relationships and acquiring positive social support are basic psychological needs of human beings. Therefore, individuals may experience loneliness if their need to belong is not effectively met [1]. As illustrated in abundant literature, loneliness is defined as a negative emotional experience caused by one’s dissatisfaction with the quantity or quality of social relationships [2]. It is worth noting that loneliness is a subjective psychological feeling, which is not equivalent to the number of interpersonal relationships possessed objectively, nor can it represent the quality of relationships.

Loneliness has become a global public health problem that seriously plagues people of different age groups [3, 4]. Loneliness is increasingly prevalent among young adults, and the negative consequences are becoming serious [5]. A survey of 46,054 participants from 237 countries and regions found that compared with middle-aged and older adults, young adults feel the highest level of loneliness, which is also more significant in males than females [6]. Gender differences in feelings of loneliness were similarly found by Pengpid et al. with the incidence of 25.1% for females and 15.0% for males [7]. In addition, in recent years, the incidence of loneliness has shown an increasing trend [8]. Social isolation, especially caused during the COVID-19 pandemic further exacerbates loneliness, and the consequential negative ramifications may last for a long time [9, 10].

Of note, loneliness, as the most common emotional experience in humans, is typically affiliated with negative outcomes. Numerous studies have shown that loneliness is significantly and positively correlated with stress, distress, anxiety, depression, anger, insomnia, self-harm, and suicidal ideation [11–14]. Meanwhile, loneliness is significantly negatively correlated with quality of life, mental health, happiness, optimism, hope, and self-efficacy [4, 15–17]. In addition, individuals with high loneliness are more likely to adopt poor behaviors and lifestyle habits, and thus threatening their health status [18]. In previous studies, loneliness was found to be a predictor of health risk behaviors(i.e., smoking, illicit drugs, using marijuana, alcohol consumption, binge drinking, and sexual risk behavior) [19].

Precisely due to the negative consequences of loneliness on health and well-being being overemphasized in most of the literature and media coverage, the rejection, denial, fear, and even stigmatization of loneliness are provoked subsequently [20]. Research in the field of stigma has shown that stigma is prevalent in different groups of people or organization [21–23]. Loneliness, as a common psychological feeling, is possible to become the target of stigma, and people who feel lonely may be treated unjustly due to it. Overall, people with high levels of loneliness are susceptible to more negative evaluations [24]. People tend to associate loneliness with the traits of weakness, unpopularity, lack of social skills, maladjustment, low achievement, apathy, and incompetence [25, 26]. In addition, even in childhood, the stigma of loneliness has been gradually formed and developed. Rotenberg et al. found that children may stigmatize and reject lonely peers, which can harm the development of self-esteem in children who feel lonely [27].

Although the stigma of loneliness and loneliness are closely related, they are not the same construct. The stigma of loneliness primarily reflects individuals’ negative attitudes and negative evaluations of loneliness [28]. People with high levels of loneliness normally have a higher stigma of loneliness, may perceive a stronger stigma when they emerge themselves in loneliness, and tend to conceal their loneliness from others [25]. Moreover, stigma exacerbates the negative implications of loneliness and reduces the likelihood that individuals who feel lonely reconnect with others [29]. Moreover, stigma exacerbates the negative implications of loneliness and reduces the likelihood that individuals who feel lonely reconnect with others [29]. Because stigma has a potentially negative impact on an individual’s social identity and status, it hinders people’s disclosure of loneliness and reduces their willingness to seek help [30]. Under the influence of stigma, even if individuals experience a strong sense of loneliness, they tend to adopt a strategy of concealment, rarely take the initiative to seek help, and may even refuse the company of others to avoid being labeled as vulnerable [31]. Meanwhile, driven by impression management motives, individuals with high loneliness stigma are less likely to reveal their loneliness to others to maintain a favorable image [32]. Although the prevalence of loneliness among young people is lower than among adults and older adults, the stigma of loneliness is particularly sever in the young population [24, 33]. Therefore, it is necessary to measure and identify loneliness stigma in young people to better develop interventions.

When the stigma of loneliness is discussed and studied, researchers have mostly adopted a qualitative approach or non-standardized assessment tools [20]. For example, in the qualitative study of stroke survivors conducted by Yang et al.‘s, it was found that individuals with high self-stigma of loneliness subjectively perceived others as holding negative views on loneliness and thus felt embarrassed and ashamed when seeking help [31]. In the study of Barreto et al., a self-administered questionnaire was used to measure individuals’ perceived stigma in the community as well as the shame associated with loneliness [25]. In that study, the questionnaire did not follow the scale development process, nor was it tested for reliability and validity, so the validity of the scale was not clarified. Moreover, in Lau et al.‘s study, participants were required to rate the target population in terms of psychological adjustment, achievement, competence, and sociability after reading a short vignette describing lonely or non-lonely college students [29]. However, this method does not apply to the rapid assessment and large-scale investigation of the stigma of loneliness.

It was evident according to the analysis of existing literature that there is a lack of valid scale for assessing the stigma of loneliness. In this regard, Ko et al. developed the Stigma of Loneliness Scale (SLS) among American college students [28]. Based on the results of open-ended questionnaires and expert opinions, an initial questionnaire consisting of 45 items was developed. After exploratory factor analysis, some items were deleted and a formal scale was then formed with 10 items in two dimensions, which were used to measure the public stigma and self-stigma of loneliness among college students. In particular, public stigma results from an individual’s stereotype, prejudice, and discrimination against loneliness. Specifically, it is the individual’s negative perception of loneliness as well as the corresponding passive emotional and behavioral responses. Moreover, Self-stigma is formed through an individual’s awareness, agreement, and application of stigma.

In Ko et al.‘s study, Cronbach’s alpha coefficients for the total scale and dimensions of SLS were observed to range from 0.73 to 0.88, and the retest reliability ranged from 0.67 to 0.68. The cross-gender invariance of SLS was verified in the cross-group test. Furthermore, the results of the incremental validity test presented a discrepancy between the stigma of loneliness and loneliness in the aspect of psychological concepts. The results of the study suggest that SLS has good reliability and validity and can be used as an effective tool to assess loneliness stigma. Moreover, SLS was significantly and positively related to loneliness, shame, self-concealment, depression, and contingent self-worth based on approval from others; while significantly and negatively correlated with social connectedness, distress disclosure, self-esteem, and social self-efficacy, both of which suggested that the effect of the stigma of loneliness on interpersonal relationships, self-awareness, and emotional experience can be predicted.

As depicted in existing literature, the SLS has not been revised to other language versions. Furthermore, there is no valid instrument available for assessing the stigma of loneliness in China, which hinders the progress of studies on the stigma of loneliness. To this end, this study was designed to translate the SLS into Chinese and examine its reliability and validity in the Chinese social context, thus providing an efficient and valid tool for assessing the stigma of loneliness among Chinese people. Considering that there are significant gender differences in loneliness and other types of stigma [9, 46], the study further examined the cross-gender invariance of SLS to better explain the underlying gender differences in loneliness stigma, and to avoid the interference caused by the inequality of measurement tools.

The study also investigated the UCLA Loneliness Scale (ULS-8), Distress Disclosure Index (DDI), Revised Cheek and Buss Shyness Scale (RCBS), Self-Concealment Scale (SCS), Social Interaction Anxiety Scale (SIAS), Social Phobia Scale (SPS), Kessler Psychological Distress Scale (K10), and Rosenberg self-esteem scale (RSES) for testing the criterion validity of the SLS. It was hypothesized that the SLS would be significantly positively correlated with the ULS-8, SS, SCS, SIAS, SPS, and K10, and negatively correlated with the DDI, and RSES.

Two factors were taken into considerations while selecting the criterion tools. On the one hand, the current study aimed to replicate the study of Ko et al. among Chinese college students, so the scale was basically the same as that of the study. In his study, it was found that there was a significant correlation between SLS and each criterion tool. Of note is that the criterion tools selected in this study are not completely consistent with the original study. For instance, RCBS was selected as the scale to evaluate college students’ shyness, while the original study used the Personal Feelings Questionnaire to measure shame. The reason for this adjustment is that the RCBS was developed specifically to assess shyness and can better reflect the concept of shyness. At the same time, the Chinese version of RCBS was revised in the college student population with good reliability and validity [41]. On the other hand, the selection of criterion tools complies with previous study results on the stigma of loneliness, in which stigma associated with loneliness is mainly reflected in lack of social skills, avoidance of social interactions, high self-concealment, low self-evaluation, and negative mental health status [24, 26, 29].

## Methods

### Translation process

The translation work was completed following five steps [42]. First, independent translations were performed by 2 translators after obtaining permission from the authors of the original scales. The first translator possessed a background in both English and psychology studies, with an undergraduate major in English and a master’s degree in mental health education. The other translator, an associate professor of English major, took on the role of “native translator”. This translator had no experience in psychology and did not understand the concept of the stigma of loneliness, by which the translator can be less influenced by an academic goal in translation. Secondly, one associate professor of psychology and two translators discussed and agreed on the inconsistent parts of the translation that were contradictory to each other. Thirdly, the Chinese version of the scale negotiated by the three persons was back-translated by an English major graduate student who had not been exposed to the original scale. The content of the items reflected in the back-translated version did not differ substantially from the original scale. Fourthly, one professor of psychology and two associate professors of psychology (all with research experience in scale development or revision) were invited to form an expert committee to comment on the translated scale. According to the experts’ opinions, some items were revised to improve the conciseness, comprehensibility, and accuracy of the scale. Fifth, 17 college students were pre-surveyed and invited to fully discuss their feelings when they responded to the scale, comprehensibility, and any ambiguity, and then fine-tuned some items. After the above five steps, the translation of the scale was completed. The translated scale was aligned with the original scale in terms of the number of items and scoring methods.

### Participants

The minimum sample size required was estimated before starting the survey. Generally, to ensure the stability of the results of factor analysis, the minimum sample size should reach 300 and more than 10 or even 20 times [43, 44]. Therefore, in this study, the minimum sample size was set at 300 for both phases. Meanwhile, informed consent was obtained from the participants, all of whom were voluntary and older than 16 years old. Participants viewed a detailed informed consent form to understand the purpose of this survey and the use of data. For subjects under the age of 18, the requirement of communication with their parents by phone or WeChat before participating in the survey, and the acquisition of their parents’ informed consent are premised. After data retrieval, the researcher removed data with identical positive and negative options and response times of less or more than 2 standard deviations. Of note, this study followed the Declaration of Helsinki and was also approved by the Ethics Committee of Jilin International Studies University (Approval number: JY202211003).

The current study conducted data investigation in two stages. As statisticians suggested, in confirmatory factor analysis, a new sample population is required to examine again the rationality of the factor structure determined in exploratory factor analysis [45]. Moreover, the majority of researchers adopted 2 or more samples to do their surveys in previous studies of scale development or revision [46–49]. Therefore, the data of the first phase as sample 1 were used to do item analysis and exploratory factor analysis; the data of the second phase as sample 2 were used to do confirmatory factor analysis and analysis of reliability and validity.

Both phases of the survey were conducted by convenience sampling for subject recruitment. The subjects came from three universities, one comprehensive university, one language university, and one Chinese medicine university, respectively, and the principal majors included education, preschool education, Chinese medicine, clinical medicine, nursing, acupuncture and massage, English, and translation. A total of 703 people were surveyed in the first stage, with 657 valid questionnaires and an effective rate of 93.46%. Of them, 211 (32.12%) were male, and 446 (67.88%) were female; 354 (53.88%) were first-year students, 205 (31.20%) were second-year students, 79 (12.03%) were third-year students, and 19 (2.89%) were fourth-year students; 434 (66.06%) lived in urban areas and 223 (33.94%) lived in rural areas; 586 Han Chinese (89.19%), 71 ethnic minorities (10.81%); 365 only children (55.56%), 292 non-only children (44.44%). The minimum age was 17 years old; the maximum age was 24 years old, and the average age was 19.80 years old (SD = 1.39).

In the second stage, 863 people were surveyed, with 801 valid questionnaires and an effective rate of 92.82%. Here, 223 (27.84%) were male and 578 (72.16%) were female; 332 (41.45%) were undergraduates and 469 (58.55%) were graduate students; 110 (13.73%) were freshmen, 47 (5.87%) were sophomores, 143 (17.85%) were juniors, 32 (4.00%) were seniors, 195 (24.34%) were first-year graduate students, 192 (23.97%) were second-year graduate students and 82 (10.24%) were third-year graduate students; 489 (61.05%) living in urban areas, 312 (38.95%) in rural areas; 737 (92.01%) Han Chinese, 64 (7.99%) ethnic minorities; 376 (46.94%) only children, and 425(53.06%) non-only children. The minimum age was 18 years old; the maximum age was 39 years old, and the average age was 23.34 years old (SD = 3.21).

### Instrument

#### Stigma of loneliness scale(SLS)

The SLS was developed by Ko et al. in 2022 and was primarily designed to assess the stigma associated with loneliness [28]. It consists of ten items and is divided into two dimensions Self-Stigma of Loneliness (SSL) and Public Stigma of Loneliness (PSL). The scale is scored on a 5-point Likert-type scale (1 = strongly disagree; 5 = strongly agree). The sum of the scores of each item is the total score, and the higher the score, the higher the level of SSL and PSL.

#### UCLA loneliness scale (ULS-8)

The ULS-8 is a brief version of the questionnaire developed by Hays et al. based on the ULS-20 [50]. Its Chinese version was demonstrated to have good reliability and validity [51, 52]. The ULS-8 consists of 8 items with a one-dimensional structure using a 4-point Likert-type scale ranging from 1(never) to 4(always). Item 3 and item 6 are reverse scoring questions. The sum of the scores of each item is the total score, and the higher the score, the higher the level of loneliness of the individual. The Cronbach’s alpha coefficient of the scale in this study was 0.855.

#### Distress disclosure index (DDI)

Developed by Kahn et al. in 2005, the DDI is primarily used to assess an individual’s tendency to hide or reveal psychological distress [53]. The validity of the Chinese version of the scale has been validated in different populations [54, 55]. The DDI consists of 12 items and is one-dimensional in structure. The scale is scored on a 5-point scale (1 = strongly disagree; 5 = strongly agree). Item 2, item 4, item 5, item 8, item 9, and item 10 were reverse scored. The sum of the scores for each item is the total score, with higher scores indicating a stronger willingness to disclose psychological distress. The Cronbach’s alpha coefficient for the scale in this study was 0.895.

#### Revised cheek and buss shyness scale (RCBS)

The RCBS developed by Cheek and Buss can be used to assess the sensitivity, discomfort, and shyness experienced by individuals in unfamiliar situations [56]. The validity of the Chinese version of RCBS in undergraduates has been tested [41]. The RCBS consists of 13 items in a single-dimensional structure. The scale is scored on a 5-point scale (1 = strongly disagree; 5 = strongly agree). Item 3, item 6, item 9, and item 12 are reverse scored. The sum of the scores for each item is the total score, with higher scores indicating higher levels of shyness. The Cronbach’s alpha coefficient of the scale in this study is 0.891.

#### Self-concealment scale (SCS)

The SCS which was developed by Larson and Chastain in 1990 was primarily designed to assess an individual’s tendency to conceal negative or distressing information about themselves from others [57]. The Chinese version of SCS has good reliability and validity [58]. The SCS consists of 10 items and is one-dimensional in structure. The scale is scored on a 5-point scale (1 = strongly disagree; 5 = strongly agree). The sum of the scores for each item is the total score, and the higher, the score the higher the level of self-concealment of the individual. The Cronbach’s alpha coefficient of the scale in this study was 0.910.

#### Social interaction anxiety scale (SIAS) and social phobia scale (SPS)

The SIAS and SPS for measuring the level of anxiety and fear in social interactions were developed by Fergus et al. in 2012 [59]. The Chinese versions of SIAS and SPS serve as effective tools in evaluating social anxiety and phobia in undergraduates [60]. SIAS and SPS consist of 6 items each and use a 5-point Linkert-type scale ranging from 1 (strongly disagree) to 5 (strongly agree). Scores of each item are summed to a total score, and the higher the score the higher the level of social anxiety and social fear of the individual. The Cronbach’s alpha coefficients of SIAS and SPS in this study were 0.924 and 0.925, respectively.

#### Kessler psychological distress scale (K10)

The K10 that was developed by Kessler et al. is available for the screening of the mental health status of residents [61] and the Chinese version has good reliability and validity [62]. The scale consists of 10 items, which are divided into two dimensions: anxiety and depression. The scale is scored on a 5-point scale (1 = all of the time; 5 = none of the time). The sum of the scores for each item is the total score, and the higher the score the riskier an individual is for developing mental illness. The Cronbach’s alpha coefficient of the scale in this study is 0.967.

#### Rosenberg self-esteem scale (RSES)

The RSES represents the most frequently employed assessment tool in self-esteem research [63]. The RSES consists of 10 items with one-dimensional structure and the Chinese version of RSES is a valid tool to measure self-esteem among Chinese people [64]. The scale is rated on a 4-point scale (1 = strongly disagree, 4 = strongly agree). Item 3, Item 5, Item 8, Item 9, and Item 10 are reverse scored. The sum of the scores for each item is the total score, with higher scores indicating higher levels of individual self-esteem. The Cronbach’s alpha coefficient of the scale in this study is 0.908.

### Statistical analysis

The collected data were analyzed using SPSS 20.0 and AMOS 24.0. First, item analysis was conducted on the data from Sample 1 to examine the discrimination and homogeneity of the items on the scale. The methods adopted included the critical ratio value, item-total correlation, and Cronbach test [65]. Second, the exploratory factor analysis (EFA) was conducted on the data from Sample 1 to examine the factor structure of the SLS. In EFA, in which factor extraction was performed using principal axis factor analysis, oblique rotation was done using the Promax method to determine the number of factors based on eigenvalues larger than one and scree plot [45, 66]. Third, the confirmatory factor analysis (CFA) was conducted on the data of sample 2 to verify the rationality of the two-factor structure. Meanwhile, the study further constructed the one-factor model and the two-factor orthogonal model as competition models to test whether the two-factor oblique structure model was the optimal model. In CFA, fitting indicators were adopted as follows, which were root mean square error of approximation (RMSEA), standardized root mean square residual (SRMR), comparative fit index (CFI), incremental fit index (IFI), Tucker-Lewis index (TLI), parsimonious normed fit index (PNFI), and parsimonious comparative fit index (PCFI). The χ2/df < 3, RMSEA < 0.08, SRMS < 0.05, CFI, IFI, TLI > 0.90, PNFI, and PCFI > 0.50 were regarded as the criteria for good model fitting [67].

Fourth, data from Sample 2 were analyzed by Pearson correlation analysis to examine the criterion validity of the SLS. Fifth, the study calculated Cronbach’s alpha coefficient to explain the reliability of the scale. A reliability coefficient value greater than 0.70 was considered a criterion of good reliability [68]. Sixth, to examine the equivalence of SLS among men and women, multiple group analysis was conducted on the data of sample 2. The study constructed Configural Invariance Model(M1), Weak Invariance Model (M2), Strong Invariance Model (M3), and Strict Invariance Model (M4) respectively [69]. The M1 model allows all parameters to be estimated freely. The M2 model sets the factor loadings to be equal for men and women. The M3 model is based on M2 and further sets the intercept equal. The M4 model proceeds to add residual equality on top of the equal factor loadings and intercepts. In the comparison between M2 with M1, M3 with M2, and M4 with M3, if both ∆CFI and ∆RMSEA are less than 0.01. It indicates that SLS has cross-gender consistency [70].

## Results

### Item analysis

The results of the item analysis are shown in Table 1. The total SLS scores were ranked from low to high, and the first 27% were taken as the low group and the last 27% as the high group. Independent samples t-test was used to examine the variability of each item. As the results revealed, the high subgroup scores of each SLS item were significantly higher than the low subgroup, with critical ratio values ranging from 26.66 to 33.34. Results of the correlation analysis indicated that the correlation between each item and the total score was significant (*p* < 0.001), with coefficient values ranging from 0.76 to 0.82, all of which were higher than the standard of 0.40. In the Cronbach test, the Cronbach’s alpha coefficient value of the total questionnaire was 0.936. If the Cronbach’s alpha coefficient value increased after deleting any item, it would indicate that the item was less homogeneous with the total questionnaire and needed to be deleted. After deleting any item, the Cronbach’s alpha coefficient of the SLS decreased in varying degrees between 0.928 and 0.931. Based on the results of the item analysis, all items were retained.

**Table 1:** The result of item analysis of SLS(*N* = 657)

Item	Low score group(*N* = 177)		High score group(*N* = 177)		t-value	Corrected Item-Total Correlation	Cronbach’s Alpha if Item Deleted
	M	SD	M	SD			
1	1.21	0.42	3.25	0.93	26.66^***^	0.76^***^	0.931
2	1.19	0.45	3.34	0.85	29.80^***^	0.80^***^	0.929
3	1.13	0.34	3.21	0.86	30.06^***^	0.79^***^	0.929
4	1.11	0.33	3.31	0.88	31.25^***^	0.81^***^	0.928
5	1.06	0.24	3.40	0.90	33.34^***^	0.80^***^	0.929
6	1.27	0.54	3.44	0.84	28.86^***^	0.80^***^	0.929
7	1.21	0.43	3.36	0.87	29.30^***^	0.82^***^	0.928
8	1.26	0.51	3.51	0.82	31.04^***^	0.81^***^	0.928
9	1.18	0.41	3.19	0.91	26.78^***^	0.79^***^	0.929
10	1.23	0.58	3.47	0.83	29.47^***^	0.78^***^	0.930

^**^p < 0.001

### Exploratory factor analysis(EFA)

Before conducting EFA, KMO, and Bartlett’s tests were performed. The results showed that the KMO value was 0.931 and Bartlett’s sphericity test value was 4881.85 (df = 45, *p* < 0.001). This indicates that the data can be subjected to EFA. The EFA results revealed (see Table 2) that a total of two factors with eigenvalues greater than 1 were extracted, with explanatory rates of 63.48% and 11.12%, respectively, and a cumulative explanatory rate of 74.60%. The factor loading values of each item ranged from 0.585 to 0.890, which was higher than 0.40, and there was no phenomenon of multiple loadings [44]; the commonality of each item ranged from 0.609 to 0.735, which was higher than the criterion of 0.40. Meanwhile, the extraction of 2 factors was found to be appropriate according to the scree plot results. In the end, all items of the SLS were retained, and they were the same as the English version of the scale in terms of dimensional division and attribution of items. Referring to the naming method of the original scale, the two factors were still named Self-Stigma of Loneliness (SSL) and Public Stigma of Loneliness (PSL), respectively.

**Table 2:** The results of exploratory factor analysis of SLS(*N* = 657)

Item	English Version	Chinese Version	SSL	PSL	C
Item 1	I would never tell another person that I am lonely because I would feel ashamed.	我绝不会告诉别人我是孤独的, 因为这会令我感到羞耻。	**0.890**	-0.084	0.691
Item 2	Being lonely would mean something is wrong with me.	孤独可能意味着我是有问题的。	**0.858** ^*^	-0.002	0.734
Item 3	If I were lonely, I would feel ashamed.	如果我是孤独的, 我会感到羞耻。	**0.817** ^*^	0.027	0.699
Item 4	I would judge myself negatively if I were lonely.	如果我是孤独的, 我会消极地评价自己。	**0.709** ^*^	0.146	0.672
Item 5	Being lonely would be embarrassing.	孤独是令人尴尬的。	**0.585** ^*^	0.246	0.609
Item 6	Others would assume that I do not have any friends if I were lonely.	如果我是孤独的, 其他人会认为我没有朋友。	0.025	**0.810** ^*^	0.686
Item 7	Others would assume that I am not very good at talking to people if I were lonely.	如果我是孤独的, 其他人会认为我不擅长与人交往。	0.107	**0.748** ^*^	0.686
Item 8	If I were lonely, others would assume that I had not made enough of an effort to not feel this way.	如果我是孤独的, 其他人会认为我没有付出足够的努力来避免这种感觉。	-0.009	**0.863** ^*^	0.735
Item 9	Others would assume it was my fault if I were lonely.	如果我是孤独的, 其他人会认为这是我的错	0.092	**0.734** ^*^	0.644
Item 10	If I were lonely, others would assume that I do not have social skills.	如果我是孤独的, 其他人会认为我缺乏社交能力。	-0.041	**0.855** ^*^	0.682

The bolded part is the factor and factor loading value where the item is located

SSL: Self-Stigma of Loneliness; PSL: Public Stigma of Loneliness; C: Commonality

### Confirmatory factor analysis(CFA)

CFA was applied to the data of sample 2 using AMOS 24.0. The results revealed that the two-factor model fitted well for each of the indices (see Table 3). To test whether the two-factor model was optimal, the study further constructed the one-factor model and the two-factor orthogonal mode as the competition model. Results suggested (see Table 3) that the one-factor model and two-factor orthogonal mode showed poor model fit indices. Therefore, the two-factor model is the optimal model.

**Table 3:** CFA and competing model fit indices(*N* = 801)^*^

Competing model	χ^2^/df	RMSEA	CFI	IFI	TLI	PNFI	PCFI	SRMR
Two-factor model	2.974	0.050	0.993	0.993	0.989	0.681	0.684	0.018
One-factor model	36.301	0.210	0.867	0.868	0.814	0.615	0.617	0.082
Two-factor orthogonal mode	22.703	0.165	0.919	0.919	0.885	0.651	0.653	0.082

^*^ RMSEA: root mean square error of approximation; CFI: comparative fit index; IFI: incremental fit index; TLI: Tucker-Lewis index; PNFI: parsimonious normed fit index; PCFI: Parsimonious Comparative Fit Index; SRMR: standardized root mean square residual

### Criterion-related validity test

The results of the correlation analysis (see Table 4) showed that the SLS, SSL, and PSL dimensions were significantly positively correlated with the total scores of ULS-8, RCBS, SCS, SIAS, SPS, and K10 (*p* < 0.01), with correlation coefficient values ranging from 0.38 to 0.66; the SLS and SSL and PSL dimensions were significantly negatively correlated with the total scores of DDI, and RSES (*p* < 0.01), with correlation coefficient values ranging from − 0.30 to -0.55.

**Table 4:** The criterion validity test of SLS(*N* = 801)^†^

	1	2	3	4	5	6	7	8	9	10	11
1.SLS	-										
2.SSL	0.93^**^	-									
3.PSL	0.94^**^	0.74^**^	-								
4.ULS-8	0.62^**^	0.54^**^	0.61^**^	-							
5.DDI	-0.37^**^	-0.30^**^	-0.39^**^	-0.51^**^	-						
6.RCBS	0.45^**^	0.38^**^	0.46^**^	0.57^**^	-0.37^**^	-					
7.SCS	0.50^**^	0.41^**^	0.51^**^	0.56^**^	-0.50^**^	0.56^**^	-				
8.SIAS	0.66^**^	0.58^**^	0.64^**^	0.62^**^	-0.39^**^	0.69^**^	0.59^**^	-			
9.SPS	0.65^**^	0.58^**^	0.64^**^	0.63^**^	-0.41^**^	0.66^**^	0.58^**^	0.87^**^	-		
10.K10	0.65^**^	0.56^**^	0.64^**^	0.71^**^	-0.46^**^	0.59^**^	0.59^**^	0.73^**^	0.74^**^	-	
11.RSES	-0.54^**^	-0.46^**^	-0.55^**^	-0.71^**^	0.55^**^	-0.54^**^	-0.52^**^	-0.55^**^	-0.56^**^	-0.65^**^	-
Mean	20.70	10.04	10.66	15.68	41.10	34.21	25.37	14.15	13.66	22.55	30.71
Standard deviation	7.66	3.95	4.25	4.56	8.20	9.12	7.79	5.19	5.16	8.58	5.47

^**^p < 0.01

^†^ SLS: Stigma of Loneliness Scale; SSL: Self-Stigma of Loneliness; PSL: Public Stigma of Loneliness; ULS-8: UCLA Loneliness scale; DDI: Distress Disclosure Index; RCBS: Revised Cheek and Buss Shyness Scale; SCS: Self-Concealment Scale; SIAS: Social Interaction Anxiety Scale; SPS: Social Phobia Scale; K10: Kessler Psychological Distress Scale; RSES: Rosenberg self-esteem scale

### Reliability test

The Cronbach’s alpha coefficients for the SLS, SSL, and PSL dimensions were 0.957, 0.941, and 0.955, respectively. The Cronbach’s alpha values of the total scale and each dimension exceeded the standard of 0.70, indicating that the internal consistency reliability of the scale was good.

### Cross-group invariance test

Multiple group analysis was used to examine the equivalence of SLS in different gender populations. The results showed (see Table 5) that the M1, M2, M3, and M4 models fit well and could be tested for equivalence. The ∆CFI and ∆RMSEA in the comparison between M2 and M1 were − 0.0002 and − 0.003, respectively, indicating that the weak invariance model was valid; the ∆CFI and ∆RMSEA in the comparison between M3 and M2 were − 0.003 and 0.0005, respectively, indicating that the strong invariance model was valid; the ∆CFI and ∆RMSEA in the comparison between M4 and M3 were − 0.022 and 0.018, both greater than the criterion of 0.01. The strict invariance model was not valid, indicating that the residuals of some of the items may differ across the genders.

The study used an independent samples t-test to analyze the gender differences in SLS. The results showed that the total SLS score was higher in men (22.34 ± 8.12) than in women (20.07 ± 7.38), and the difference was significant (t = 3.64, *p* < 0.001); the SSL score was higher in men (10.88 ± 4.27) than in women (9.71 ± 3.78), and the difference was significant (t = 3.59, *p* < 0.001); PSL scores were higher in males (11.46 ± 4.52) than in females (10.36 ± 4.11) and the difference was significant (t = 3.16, *p* = 0.002).

**Table 5:** Results of cross-gender invariance analysis of SLS(*N* = 801)^*^

Model	χ^2^/df	CFI	IFI	TLI	PNFI	PCFI	SRMR	RMSEA (90%CI)	∆CFI	∆RMSEA
M1	3.67	0.981	0.981	0.972	0.671	0.676	0.032	0.058 (0.050–0.066)		
M2	3.39	0.981	0.981	0.975	0.757	0.763	0.036	0.055 (0.047–0.062)	<0.001	-0.003
M3	3.43	0.978	0.978	0.975	0.861	0.869	0.037	0.055 (0.048–0.062)	-0.003	<0.001
M4	5.28	0.956	0.956	0.956	0.946	0.956	0.063	0.073 (0.067–0.080)	-0.022	0.018

^*^ M1, Configural Invariance model; M2, Weak Invariance model; M3, Strong Invariance model; M4, Strict Invariance model. RMSEA: root mean square error of approximation; CFI: comparative fit index; IFI: incremental fit index; TLI: Tucker-Lewis index; PNFI: parsimonious normed fit index; PCFI: Parsimonious Comparative Fit Index; SRMR: standardized root mean square residual

## Discussion

After translating the SLS into Chinese, a two-stage investigation was conducted to examine its reliability and validity. When the item analysis, EFA, CFA, criterion validity test, reliability analysis, and cross-group invariance analysis were conducted, it was shown that the Chinese version of SLS has satisfactory psychometric properties. The revision and validation of the Chinese version of SLS provides a scale with good reliability for assessing the stigma of loneliness in Chinese young people. The Chinese version of the SLS contributes to the maintenance of young people’s health by offering instrumental support for the development of relevant clinical practices.

The results of EFA and CFA indicated that the two-factor structure of stigma of loneliness is the optimal model compared to other factor structures. In most cases, stigma is divided into public stigma and self-stigma [71]. The stigma of loneliness may come from both external and internal sources, manifesting social and individual denial and rejection of loneliness [72]. The public stigma of loneliness mainly reflects the individual’s negative evaluation of loneliness and its adverse outcomes. For instance, loneliness is perceived as shameful, embarrassing, and problematic. Self-stigma, on the other hand, is an individual’s awareness of negative public attitudes toward loneliness based on his or her own experience of loneliness, which is identified with and incorporated into self-concept. In addition, the analysis of the criterion validity both provides empirical evidence to explain the validity of the SLS and enriches understanding of the stigma of loneliness.

In previous studies, it was found that males and females do not have an identical understanding of public stigma and self-stigma [73, 74]. There are many reasons behind the gender difference, among which the inequality of measurement tools is one of the potential factors. Potential discrepancies are only interpreted as real gender differences if the measurement instruments are equivalent to the different gender groups. Equivalence implies that males and females use the same criteria and ways to comprehend the scale items. For this reason, the study further examined the cross-gender invariance of SLS. It was found that the Configural Invariance model, Weak Invariance model, and Strong Invariance model are all valid, while the Strict Invariance model is not. Residual equivalence, as the strictest equivalence restriction, is difficult to be satisfied in most studies and is not mandatory regarding testing differences in factor structure or latent means [75]. Generally, the valid Strong Invariance model is sufficient to prove the scale’s cross-group consistency [76], which indicates that the Chinese version of SLS can be analyzed for gender differences.

The study found that men scored significantly higher than women in the SLS total score and the SSL and PSL dimensions. That is, women had lower levels of public stigma and self-stigma of loneliness compared to men. The findings of previous studies on gender differences in public stigma and self-stigma are inconsistent. In the area of public stigma, most of the literature supports that men perform higher levels of public stigma than women [74, 77]. That coincides with the result of the current study regarding to stigma of loneliness, where men were the target of intervention. Typically, women have higher levels of compassion and sympathy, which contribute to less public stigma [78].

Previous studies on self-stigma have not concluded in the same way. For instance, in a study by Barreto et al., it was found that females had higher levels of self-stigma of loneliness than males, while men had higher levels of public stigma of loneliness, were more sensitive to stigmatizing information, and were more strongly affected by it [25]. However, Pfeiffer et al. found that there was no gender difference in self-stigma [79]. The result of the current study does not share the same pattern as the above two studies. Topkaya et al. discovered that men enjoy significantly higher levels of both public stigma and self-stigma than women [80], which corresponds with current results.

Barreto et al. explained the higher self-stigma in women than in men as a result of women’s stronger sense of perception of self-stigma than men. However, the measurement was a non-standardized scale in Barreto et al.‘s study, and was conducted in a Western individualistic culture. In contrast, the current study was explored in a Chinese social context, and cultural differences may be one of the reasons for the different findings. The interdependent self is predominant in the Chinese’s self-concept, and they value interpersonal relationships the most [75]. The Chinese incorporate family, relatives, friends, and other relationships into the integrality of self and construct a complex relational network according to the degree of closeness and distance [82]. To this end, a general attitude of denial and sympathy toward loneliness and the higher levels of stigma grow among the Chinese. No matter whether males or females, the Chinese are quite sensitive to information associated with the stigma of loneliness. Furthermore, to a large extent, self-stigma stems from the perception, identification, and application of public stigma [83]. Considering that men have a higher level of the public stigma of loneliness, the more they are exposed to stigma information, the more they intend to develop intensive self-stigma of loneliness.

The study is of important theoretical value. Based on the analysis of the existing literature, it is clear that this study is the first to revise the SLS in the context of Chinese society, which provides instrumental support for a better understanding of the stigma of loneliness in Chinese. In addition, the study can be seen as a re-exploration and verification of the psychological structure of loneliness stigma. In most cultures, people who feel lonely are more likely to be labeled negatively and then stigmatized [31]. However, different cultures do not share identical views and attitudes toward loneliness, which makes sense for certain cultural differences in the stigma of loneliness. It has been noted that loneliness is less tolerated and more intensely stigmatized in countries with predominantly collectivist cultures compared to individualistic cultures [25]. China is a typical collectivist country, emphasizing family and social relations [84]. In most cases, Chinese people see themselves as a member of a group or part of a relationship, and value bonding with others and seeking social support [85]. In China, not only loneliness is frequently regarded as a negative emotion, but also people who feel lonely are more likely to be considered to have a deficit in social ability and responsibility [86]. Therefore, there exists a potential discrepancy in the understanding of loneliness between Chinese and Americans due to cultural differences. As shown in the current study, the SLS is equipped with cross-cultural applicability to some extent, which, therefore, provides an effective tool for the development of cross-cultural comparative research on the stigma of loneliness.

This study also has certain practical value. Loneliness stigma like loneliness is also prevalent in different populations and may threaten an individual’s social interaction and mental health. The current research can provide a rapid screening tool to assess the situation of loneliness stigma, its influencing factors, key populations, and the effectiveness of interventions. In addition, the analysis of criterion validity suggests that lonely people tend to have higher loneliness stigma and lower willingness to disclose themselves. Lonely people, being affected by stigma, may conceal their loneliness to avoid being negatively evaluated. Therefore, when intervening on loneliness, it is necessary to pay attention to the individual’s attitude and comments towards loneliness. Schools should consider to include interventions of contact, education, and protest, in psychology related knowledge dissemination or activities, to reduce the stigma of loneliness. In addition, in consultation, counselors can assess the stigma of individuals with high levels of loneliness, emphasizing that loneliness is a normal and pervasive experience to increase motivation of social re-connection.

### Limitations and future research

There are several limitations in this study. First, the study adopted a convenient sampling method to recruit participants from only 3 universities, which may have caused sampling bias. The number of females in this study was significantly higher, while males only accounted for approximately 1/3 of the total number. Meanwhile, the students’ majors were mainly medicine, education, and linguistics, and there was a lack of students in engineering, agriculture, management, and other majors. In future studies, a stratified random sampling method could be considered to improve the representativeness of the subjects. Second, the study mainly included Han Chinese students, which resulted in the unclear applicability of the scale among minority groups. China is a multi-ethnic country of 56 ethnic groups, mainly including Mongolians, Hui, Tibetans, Uyghurs, Miao, and etc. Different ethnic groups have diverse lifestyles and cultural concepts, and their perceptions of loneliness also differ. For example, the Han nationality mainly cultivate agrarian culture, while Mongolians mainly cultivate nomadic culture. Compared to Han Chinese, Mongolians may have a higher tolerance and acceptance of loneliness. Therefore, in future studies, the applicability of SLS can be further tested among different ethnic groups and the role cultural factors played in the formation, development, and intervention of the stigma of loneliness can be analyzed.

Third, loneliness is prevalent in different age groups, especially in older adults who are threatened by illness and death, resulting in higher levels of loneliness. There is evidence that attitudes toward loneliness are not consistent across age groups [34]. Therefore, whether SLS is equivalent among young, middle-aged, and older populations still needs further validation. Moreover, future studies can develop specialized assessment tools based on the psychological characteristics of different age groups. Fourth, the English version of SLS was developed in 2022, which has not been widely used during the relatively short time. In addition to this study, SLS has not been revised into other languages. Therefore, the validity and cross-cultural applicability of the scale still require more empirical studies to test. Moreover, as Ko et al. pointed out, adopting a self-reporting approach may not capture the full implications of loneliness stigma. This study did not address these limitations. The study only translated SLS into Chinese and tested the reliability and validity among Chinese undergraduates, and did not add other items. Therefore, the Chinese version of the scale may also not fully meet the understanding of loneliness stigma among Chinese college students.

## Conclusion

The results of the current study showed that the Chinese version of SLS consisted of 10 items, which are divided into two dimensions: public stigma of loneliness and self-stigma of loneliness. The Chinese version of SLS is consistent with the English version in terms of the number and attribution of items and has been validated in terms of the rationality of the two-factor structure in the Chinese social context. The results of the reliability and validity analysis demonstrated that the Chinese version of the SLS has satisfactory psychometric properties and can be used as a valid tool for assessing the stigma of loneliness among Chinese people. The results of the study present a reference for the development of research and intervention practices on the stigma of loneliness, which are embedded with clinical value in reducing the negative effects of loneliness.

## Data Availability

The datasets generated and/or analyzed during the current study are not publicly available due to Chinese people being relatively secretive about their lives and thoughts, although informed consent was obtained from study subjects before the survey. The findings were largely reported and are available from the corresponding author on reasonable request.

## Abbreviations

CFA: Confirmatory factor analysis
CFI: Comparative fit index
DDI: Distress disclosure index
EFA: Exploratory factor analysis
IFI: Incremental fit index
K10: Kessler psychological distress scale
M1: Configural invariance model
M2: Weak Invariance model
M3: Strong Invariance model
M4: Strict Invariance model
PCFI: Parsimonious comparative fit index
PNFI: Parsimonious normed fit index
RCBS: Revised cheek and buss shyness Scale
RMSEA: Root mean square error of approximation
RSES: Rosenberg self-esteem scale
SCS: Self-concealment scale
SIAS: Social interaction anxiety scale
SLS: Stigma of loneliness scale
SPS: Social phobia scale
SRMR: Standardized root mean square residual
TLI: Tucker-lewis index
ULS-8: UCLA loneliness scale;
